# Vitamin D status in Greenland – dermal and dietary donations

**DOI:** 10.3402/ijch.v72i0.21225

**Published:** 2013-08-05

**Authors:** Stig Andersen, Anna Jakobsen, Hanne Lynge Rex, Folmer Lyngaard, Inge-Lise Kleist, Peder Kern, Peter Laurberg

**Affiliations:** 1 Arctic Health Research Centre, Aalborg University Hospital, Aalborg, Denmark; 2 Department of Internal Medicine, Queen Ingrids Hospital, Nuuk, Greenland; 3 Department of Geriatric Medicine, Aalborg University Hospital, Aalborg, Denmark; 4 Department of Gynaecology and Obstetrics, Queen Ingrids Hospital, Nuuk, Greenland; 5 Department of Endocrinology, Aalborg University Hospital, Aalborg, Denmark

**Keywords:** vitamin D, diet, lifestyle changes, UVB radiation, obesity, Inuit Eskimos, Arctic Greenland, review

## Abstract

**Background:**

Vitamin D status influences skeletal health, the risk of falls and fractures, and muscle health, and it has been associated with inflammatory, infectious, cardiovascular and metabolic disorders in addition to some cancers. Prevailing intracellular infections such as tuberculosis are speculated to relate to vitamin D status. The vitamin D sources are dietary and dermal, the latter depending on UVB radiation exposure from the sun. Life in the Arctic influences vitamin D status because of dietary peculiarities, the polar night, waning of the ozone layer and maybe ethnic differences between Inuit and non-Inuit.

**Objective and design:**

Data on vitamin D status as estimated by plasma 25OHD in Inuit and non-Inuit in Greenland are reviewed.

**Results:**

Decreasing intake of vitamin D-rich local food items associated with decreasing plasma 25OHD levels and insufficient vitamin D status is seen with low intake of traditional Inuit foods. Plasma 25OHD levels increase markedly during spring and summer in parallel with the high influx of sunlight while plasma 25OHD is not influenced by obesity in Greenland Inuit and no clear-cut association is seen between plasma 25OHD and the risk of tuberculosis.

**Conclusion:**

The frequency of vitamin D deficiency in populations in Greenland rises with the dietary transition and diseases related to low vitamin D status should be monitored.

Vitamin D is important for human health. Low vitamin D is associated with adverse health outcomes as it increases the risk of osteoporosis, falls and fractures (1–6). The discovery that most tissues in the human body have vitamin D receptors encouraged epidemiological surveys that have linked low plasma vitamin D to neuropsychological functioning, a number of autoimmune diseases, immune response and infectious diseases, hypertension and cardiovascular disorders, diabetes and metabolic syndrome, and some cancers (7–17), though findings are not universal (18) and randomized controlled trials so far have failed to identify an effect of vitamin D supplementation on these diseases (19–22).

## Sources of vitamin D

Vitamin D comes from either dietary sources or it is synthesized in the skin following exposure to sunlight.

The dermal production of 25-vitamin-D depends on conversion of 7-dehydrocholesterol to previtamin D_3_ and further isomerization to vitamin D_3_. This occurs when solar ultraviolet B radiation penetrates the skin (1,8).

The number of foods rich in vitamin D is limited. However, sea mammals and free-living fish such as salmon and cod are rich in vitamin D (17,23,24). In addition, some countries have foods fortified with vitamin D (1,7).

Vitamin D from the skin and the diet is metabolized in the liver to 25-hydroxy-vitamin-D (25OHD). This is further metabolized to its active form 1,25-dihydroxy-vitamin-D in the kidney under tight control. Parathyroid hormone (PTH) stimulates 1,25-dihydroxy-vitamin-D production while fibroblast growth factor 23, which is secreted from the bone, suppresses 1,25-dihydroxy-vitamin-D production and induces the expression of 25-hydroxy-vitamin-D-24-hydroxylase. The latter enzyme converts both 25-hydroxy-vitamin-D and 1,25-dihydroxy-vitamin-D into inactive calcitropic acid, which is excreted in the urine (1,8). Thus, a diligent balance of vitamin D is maintained in the human body.

## Diet and vitamin D in Greenland

Free-living fish and sea mammals rich in vitamin D make up the traditional diet in Greenland (17,25,26) and Greenland Inuit consider seal and whale blubber, which is rich in vitamin D, to be of particular dietary value.

The Westernization of Greenlandic societies that started around 1960 has shifted the dietary habits away from the traditional Inuit food items (25,27). This dietary transition occurred at different paces in different parts of Greenland (27), so that today settlements, towns and the capital city display different degrees of Westernization (25,27). This gradual transition has been used to describe the influence of traditional Inuit diet on vitamin D in Greenland.

**Fig. 1:**
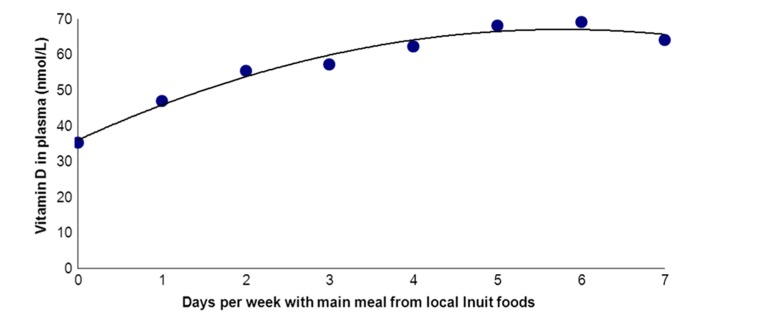
Plasma 25OHD_2+3_ (nmol/L) in participant groups split according to number of days per week with main meal from traditional food items among population groups in the capital city Nuuk (64°15'N) in West Greenland and in Ammassalik district (65°35′N) in rural East Greenland. The figure is based on data from ref. (28). Vitamin D in plasma was described (r^2^=97.5%) by: Vitamin D=36.1+10.7 day −0.92 day^2^.

Andersen and colleagues (28) assessed the importance of traditional Greenlandic food for vitamin D in 535 Inuit and non-Inuit living in the capital city Nuuk in West Greenland and in the rural Ammassalik district in East Greenland. Dietary habits were assessed in 2 ways. First, participants were split into quintiles by food frequency scores based on a detailed food frequency questionnaire (FFQ) (28). Second, participants were split by the number of days per week that they reported that the main meal was from Greenlandic food items (28) and this was validated by measuring urinary iodine excretion as a biomarker of adherence to traditional Inuit foods that are particularly rich in iodine (25). Plasma 25OHD differed markedly between the 5 FFQ-based diet groups (28). A diet based on mainly imported foods was associated with a mean total plasma 25OHD level of 36 nmol/L. This increased gradually to 68 nmol/L in the group with a diet comprising mainly traditional Greenlandic food items, and more individuals were in the range of a sufficient vitamin D level (plasma 25OHD >50–80 nmol/L). Figure 1 shows that similar differences were seen with the number of weekdays that the main meal consisted of Greenlandic food items. Interestingly, ethnicity was important to plasma 25OHD after adjusting for diet, supplements, body mass index (BMI), age, sex and place of living. Furthermore, ethnicity influenced the importance of traditional Inuit diet for plasma 25OHD (28). It was puzzling that 25OHD increased more between the lower quintile FFQ groups than between the higher FFQ quintile groups (28). This is also seen in Fig. 1.

The traditional Inuit diet includes caribou, birds, fish, seal and whale. A search for the influence of individual dietary components on plasma 25OHD in Inuit showed that seal and whale were the food items that influenced plasma 25OHD the most (28).

Some Greenland Inuit have migrated to Denmark (29) with a change of diet (30). Rejnmark et al. investigated 54 Greenlanders and 43 Caucasian Danes in Denmark for comparison with 91 Greenlanders in the capital city of Nuuk in Greenland (30). They found that Inuit eating seal or whale once a week or more had a plasma 25OHD of 53 nmol/L compared to 32 nmol/L in those who ate seal or whale less than once a week. These summer values decreased to 41 and 29 nmol/L in winter. Interestingly, they found lower plasma 25OHD in Inuit compared to non-Inuit both living in Denmark on a non-traditional diet (30).

## Sun and vitamin D in Greenland

Living in the circumpolar regions influences the exposure to UVB radiation in different ways. First, the circumpolar areas above the Arctic Circle are characterized by a winter season of no direct sun and summers of continuous sun. Second, the solar zenith angle is very high. This decreases the intensity of the UVB radiation that is absorbed through its oblique passage through the atmosphere. Third, the Arctic environment is defined by a mean temperature below 10°C during the warmest month. This influences clothing and hence dermal exposure to UVB radiation. Fourth, the thinning of the ozone layer over the polar areas may likely increase the UVB radiation in these areas (31). Fifth, hunters spend up to 24 hours outdoors daily during summer with midnight sun in North Greenland, which is a high-pressure area with a long line of sunny days and nights, during spring and summer. Sixth, reflections from snow, ice and sea add to the intensity of the light during these sunny days in spring. Seventh, Inuit have darker skin than Caucasians and thus require more UVB radiation for dermal vitamin D production. The sum of influence of these factors on dermal UVB exposure and thus production of 25OHD causes considerable uncertainties in estimates of dermal vitamin D production in circumpolar populations.

**Fig. 2:**
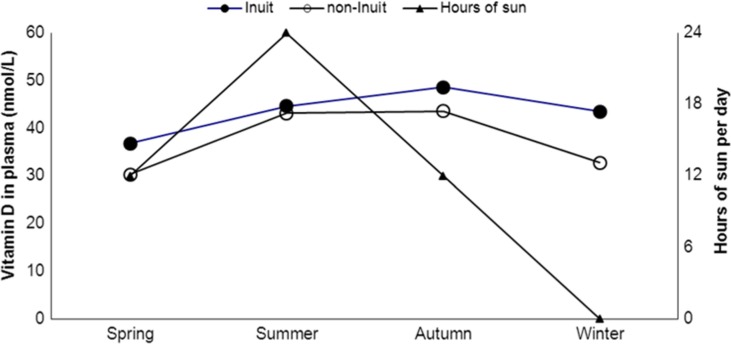
25-OH-vitamin D (nmol/L) in plasma among Inuit men and women living in the Disco Bay area around 70°N in North Greenland and the number of hours the sun is up. The polar night extends 1 month in December–January and the sun does not set for a full month in June–July. The figure is based on data from ref. (32).

Greenland hosts the most northern habitats on Earth and a study conducted in the Disco Bay area in North Greenland (70° north) included 97 Inuit and non-Inuit living 400 and 500 kilometres north of the Arctic Circle (32). People were examined and blood samples were taken at equinox, by the end of the polar night and after 1 month of midnight sun. Figure 2 illustrates that plasma 25OHD levels were higher following the sunny season, that is, the synthesizing season. Season remained important for plasma 25OHD after adjusting for diet, time spent on outdoor activities, gender, age, ethnicity, use of supplements, body weight and residence (32).

The findings correspond to that of Rejnmark et al. (30) among Greenland Inuit further to the south. They found a difference between summer and winter samples of 25OHD among 45 Inuit in Nuuk eating seal or whale less than once weekly. Their similar finding among 54 Inuit living in Denmark with an equally low intake of traditional Inuit diet suggested that the influence of season did not differ much between Nuuk in Greenland at 64° north and Denmark at 55° north (30).

These seasonal differences in vitamin D strongly suggest that dermal vitamin D production adds to plasma 25OHD among populations in central and North Greenland though the studies did not actually measure the production.

**Fig. 3:**
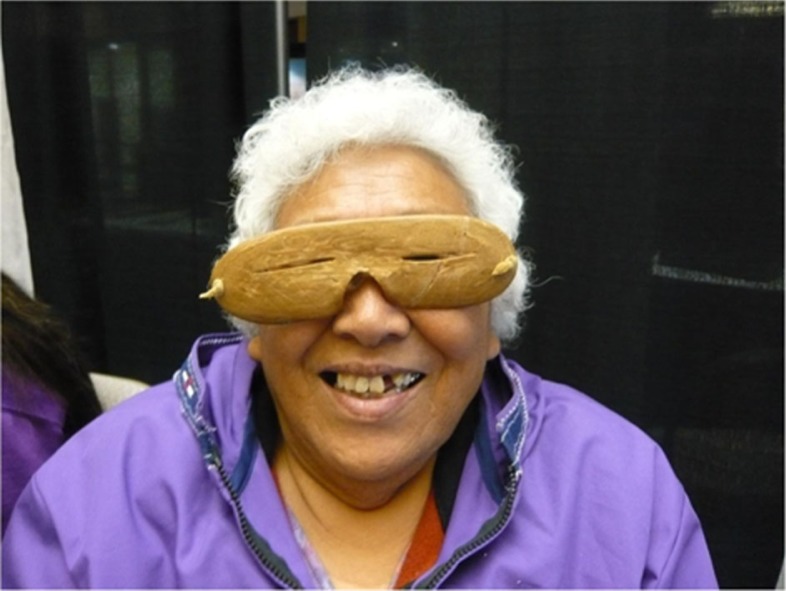
Picture showing an elderly Alaskan Inuit woman demonstrating her traditional “sunglasses” at the 15th International Congress of Circumpolar Health held in Fairbanks in August 2012. Similarly, eye protection from sealskin was known to be necessary to Inuit hunters in Greenland during spring and summer (33).

Differences between methods for measurement of UVB radiation are important. The measured UVB radiation in Greenland has increased as the spectrometer has been changed to measure UVB radiation perpendicularly to the sun rather than on a horizontal surface (31). The UVB index now goes beyond 3.5 during summer in North Greenland where hunters spend many hours outdoors during spring and summer (32) under the waning ozone layer (31). Furthermore, the reflection of sun from snow, ice and water made it necessary for Inuit hunters to wear eye protectors long before the invention of sunglasses (33). Figure 3 shows traditional eye protection worn by an elderly Inuit woman.

## Ethnicity and vitamin D in Greenland

It has been speculated that the frigid Arctic environment caused a selection of Inuit to adapt to a low calcium diet and low vitamin D (17,34). Most of the changes mentioned above are more recent and are thus not likely to counteract this hypothesized selection.

Ethnicity influenced plasma 25OHD in 2 ways in the study of the influence of diet on vitamin D (28). It had a direct influence on plasma 25OHD in the multivariate analysis, and in addition it modified the influence of traditional Greenlandic diet on plasma 25OHD (28).

The influence of ethnicity was also distinct in the study of seasonal changes in plasma 25OHD among Inuit and non-Inuit in North Greenland (32). Andersen et al. found an almost 3 times higher risk of having a plasma 25OHD below 50 nmol/L among non-Inuit compared to Inuit after adjusting for diet, season, sex, age, supplement use, body weight, and residence (32).

The study of Inuit in Nuuk also found a clear effect of ethnicity on plasma 25OHD (30) and a similar ethnic influence was found on other parameters in the calcium metabolism. Thus, a higher plasma 1,25-dihydroxy-vitamin in Inuit than in non-Inuit suggested a high 1-alpha-hydroxylase activity. Similarly, PTH and bone alkaline phosphatase differed with ethnicity (30). This supports an ethnic difference in vitamin D between Inuit and non-Inuit.

## Obesity and vitamin D in Greenland

Obesity is associated with lower plasma 25OHD in other populations (8) and obesity rates have increased markedly in parallel with the transition of societies in Greenland (35). However, there is some debate on the importance of high BMI in Inuit as the cut-off points delineating being overweight may be raised in Inuit compared to non-Inuit (35,36). This could influence the level of BMI that affects plasma 25OHD.

The study in North Greenland reported no association between body weight and plasma 25OHD, but it included only 97 individuals (32) and was not sufficiently powered to reject the hypothesis of an influence of BMI on plasma 25OHD.

**Fig. 4:**
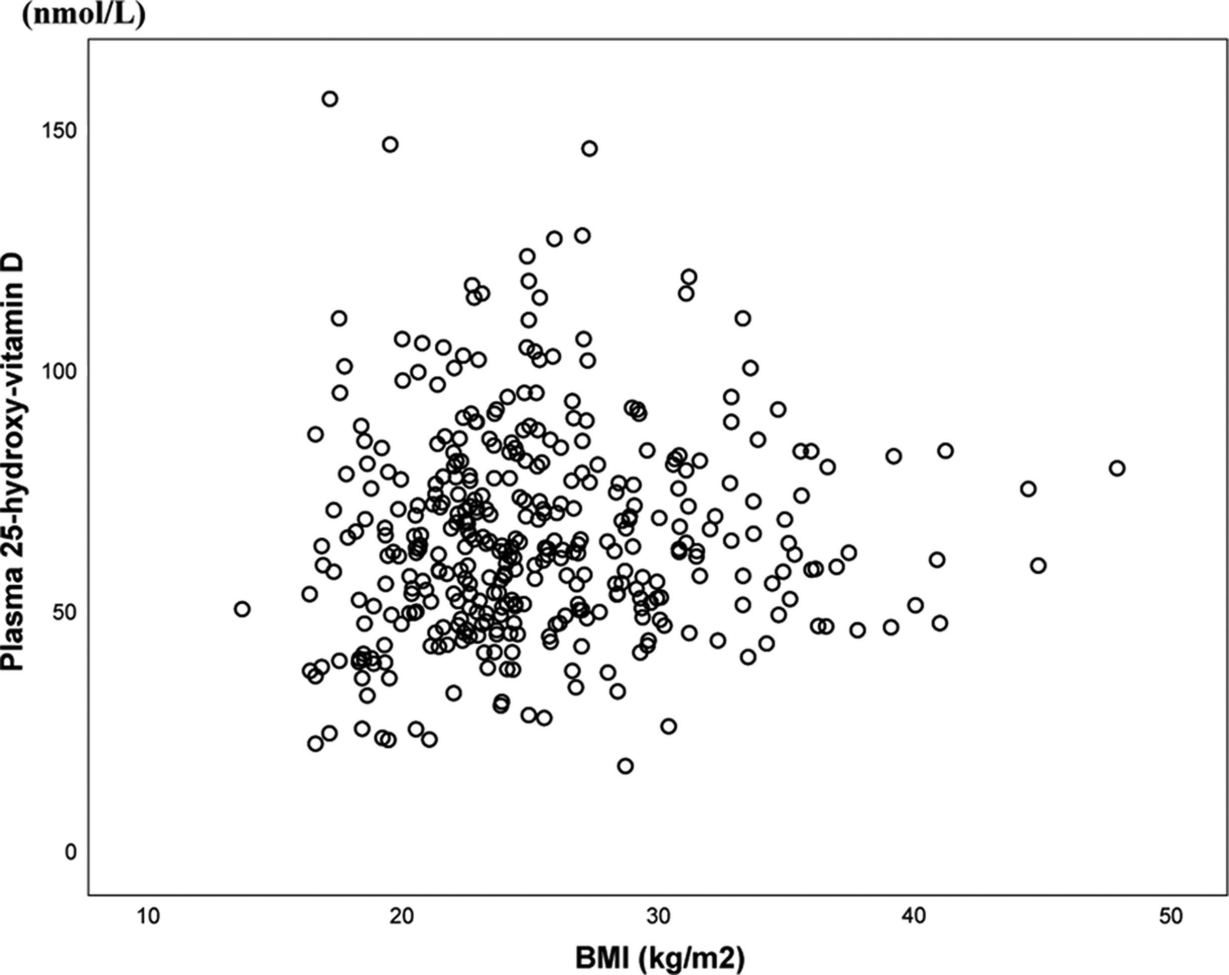
The association between body mass index (BMI) and plasma 25OHD (nmol/L) among 434 Inuit in the capital Nuuk in West Greenland and Inuit in Ammassalik district in East Greenland (r^2^= − 0.002, p = 0.68). Data from ref. (28) and ref. (35).

The study of dietary transition and vitamin D included 434 Inuit in Nuuk in West Greenland and Ammassalik in East Greenland. Figure 4 illustrates the lack of association between BMI and plasma 25OHD confirmed upon testing adjusted for age and gender (28). Thus, obesity did not influence plasma 25OHD in this population and the obesity epidemic is not a concern with respect to vitamin D in Greenland Inuit.

## Conclusions and perspective

Life in the Arctic environment poses special challenges to vitamin D homeostasis (17,37). Hunter populations have lived in Greenland for a thousand years without vitamin D supplementation. They may have adapted to the Arctic environment so that today no detrimental effects can been seen on bones or muscles (38).

Dermal vitamin D production is marked but the transition of societies in Greenland decreases the vitamin D levels, and levels categorized as insufficient are now common in Greenland (18,28,30,32). Still, the current knowledge supports an ethnic influence on vitamin D homeostasis in Greenland Inuit that needs further attention.
